# Aromatic plants as cosmeceuticals: benefits and applications for skin health

**DOI:** 10.1007/s00425-024-04550-8

**Published:** 2024-11-05

**Authors:** Jesus Olivero-Verbel, Patricia Quintero-Rincón, Karina Caballero-Gallardo

**Affiliations:** 1 Environmental and Computational Chemistry Group. School of Pharmaceutical Sciences, Zaragocilla Campus, University of Cartagena, Cartagena, 130014 Colombia; 2 Functional Toxicology Group. School of Pharmaceutical Sciences, Zaragocilla Campus, University of Cartagena, Cartagena, 130014 Colombia; 3 Research Group Design and Formulation of Medicines, Cosmetics, and Related, Faculty of Pharmaceutical and Food Sciences, Universidad de Antioquia, Medellín, 050010 Colombia

**Keywords:** Cosmeceutical, Bioactive compounds, Antioxidants, Anti-aging, Antiphotoaging, Aromatic plants

## Abstract

**Main conclusion:**

This review highlights the potential of aromatic plants as natural antioxidants in cosmeceuticals to combat skin aging and promote health and rejuvenation.

**Abstract:**

Aromatic plant extracts, essential oils, or their phytoconstituents have a long history of use in skincare, dating back centuries. Currently, these plant-based sources are extensively researched and utilized in the cosmeceutical industry to formulate products that enhance skin health and promote a youthful appearance. These plants’ diverse bioactivities and sensory properties make them ideal ingredients for developing anti-aging agents recommended for maintaining healthy skin through self-care routines, offering a natural alternative to synthetic products. Reactive oxygen species (ROS) accumulation in the dermis, attributed to intrinsic and extrinsic aging factors, particularly prolonged sun exposure, is identified as the primary cause of skin aging. Plant extracts enriched with antioxidant compounds including flavonoids, phenolics, tannins, stilbenes, terpenes, and steroids, are fundamental to counteract ROS-induced oxidative stress. Noteworthy effects observed from the use of these natural sources include photoprotective, senolytic, anti-inflammatory, anti-wrinkle, anti-acne, and anti-tyrosinase activities, encompassing benefits like photoprotection, wound healing, skin whitening, anti-pigmentation, tissue regeneration, among others. This review highlights several globally distributed aromatic plant species renowned for their benefits for skin, including *Foeniculum vulgare* Mill. (Apiaceae), *Calendula officinalis* L. and *Matricaria chamomilla* L. (Asteraceae), *Thymus vulgaris* L. (Lamiaceae), *Litsea cubeba* (Lour.) Pers. (Lauraceae), *Althaea officinalis* L. (Malvaceae), *Malaleuca alternifolia* (Maiden y Betche) Cheel (Myrtaceae), *Cymbopogon citratus* (DC.) Stapf (Poaceae), *Rubus idaeus* L. (Rosaceae), and *Citrus sinensis* L. Osbeck (Rutaceae), emphasizing their potential in skincare formulations and their role in promoting health and rejuvenation.

## Introduction

The cosmeceutical properties of aromatic plants date back to ancient times (Lall et al. 2020). These botanical species produce volatile compounds called essential oils (EOs), which are responsible for their distinctive fragrances (Mishra and Chandra 2022). Aromatic plants have significant value in various industries including perfumery, aromatherapy, food processing, and cosmetics. Also, EOs do not accumulate hazardous metals that allow entry into the food chain; therefore, they are safe for industry and favor sustainable cultivation on a large scale (Mishra and Chandra 2022).

The studies have demonstrated the immense potential of plants to treat various pathologies. Hence, extracts, essential oils, and phytocompounds of aromatic species are widely used in both the pharmaceutical and cosmetic industries as active ingredients, taking advantage of their medicinal and organoleptic properties (Michalak 2022).

Therapeutic strategies for skin care are closely linked to the presence of endogenous antioxidants (polyphenols, including phenolic acids, flavonoids, tannins, and stilbenes, as well as steroids, terpenes, carotenoids, and steroidal saponins) biosynthesized by most botanical species as a defense mechanism against external aggressors, for example, those caused by high exposure to sunlight (Quintero-Rincón et al. 2023). These biosynthesized antioxidants are appropriate for the development of cosmeceuticals because they reduce ROS damage in skin cells and modulate molecular cell receptors, gene expression, and inhibition of enzymatic activity (Hernandez et al. 2021).

The term "cosmeceutical" describes a product that exerts both cosmetic and pharmaceutical properties (Carpio et al. 2021), and whose use is oriented to pharmaceutical therapeutic benefits on the skin (Pandey et al. 2019). Draelos (2008) defines a cosmeceutical as a “functional cosmetics designed to adorn the face and body without changing the structure of the human form”. These products contain natural bioactive components, vitamins, and enzymes, that can derive from plants, marine organisms, and microorganisms (Alves et al. 2020), and have a low toxicity profile. The major therapeutic benefits obtained from cosmeceutical products are anti-aging, skin hydrating, photoprotection, depigmenting, anti-wrinkle, and anti-inflammatory, as well as their antibacterial (anti-acne) and antifungal properties, among others (Pandey et al. 2019; Alves et al. 2020).

Currently, society experiences a strong need to consume innovative products that contribute to promoting a healthy appearance and a youthful face through the use of cosmetics that include natural ingredients as part of a more natural mode of life (Lall et al. 2020; Strzępek-Gomółka et al. 2021; Michalak et al. 2021; Chaiyana et al. 2021), which has led to an exponential increase in the global demand for cosmeceutical products (Ullah et al. 2017).

The receptiveness of cosmeceutical products in the global beauty and personal care market has sparked a notable interest in both industry and academia to explore the beneficial properties of aromatic plants. This interest is reflected in the significant increase in publications on the topic, which has experienced spectacular growth in recent years.

**Fig. 1:**
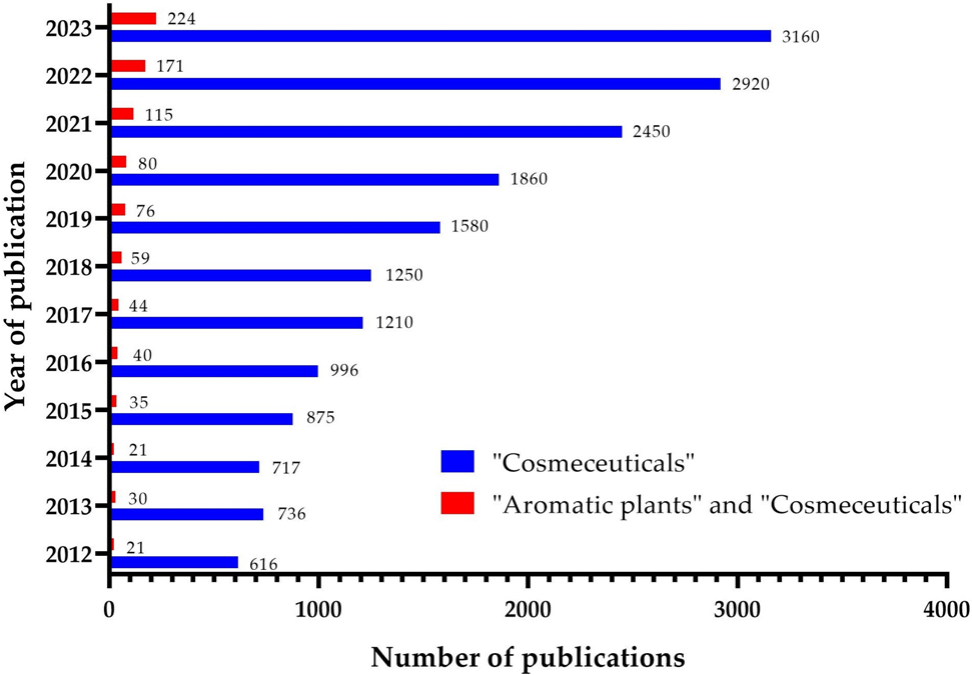
Publications on “aromatic plants” and “cosmeceuticals” for the last 12 years

A search carried out by Scholar Google (accessed in May 2024) showed information about the number of investigations related to “cosmeceuticals” and “aromatic plants” used for their beneficial properties for human health. The first publications appeared in 2003, where the benefits of plants such as *Arnica montana* L*.*, *Chamomilla recutita* L. or *Matricaria chamomilla* L., *Hamamelis virginiana* L., and *Calendula officinalis* L. were highlighted for their properties for the management of skin accesses (furunculosis), surface-phlebitis and lesions of the skin surface, astringent and hemostatic, and improvement of wound healing, respectively (Schulz 2003), including other medicinal species that are currently of commercial importance. However, in the last 12 years, there has been an increase in research related to cosmeceuticals (Fig. 1). This growing interest has been facilitated by the discovery of new technologies and the incorporation of novel natural ingredients for the promotion of healthy skin. In this way, a total of 18,730 articles were published, of which 916 specifically are focused on the cosmeceutical properties of aromatic plant species, which account for approximately 5% of the most extensively studied natural sources, especially for their anti-aging and antiphotoaging properties.

Plant-based cosmeceuticals are increasingly recognized for their ability to improve skin health. Compared to synthetic alternatives, these natural products exhibit superior skin tolerance and efficacy and serve as a renewable and sustainable source of active compounds. Furthermore, plant-based cosmeceuticals are more affordable, making them accessible to consumers (Goyal et al. 2022).

To mitigate potential adverse reactions associated with prolonged use of chemical-based synthetic cosmetics, the industry employs extracts and EOs rich in antioxidant properties as viable ingredients. Numerous studies have shown that the chemical composition of these natural ingredients can effectively reduce oxidative stress, presenting innovative alternatives for cosmeceutical formulations (Prasanth et al. 2019). In this context, a literature review was conducted to determine the benefits for the skin of some aromatic plants. The review aims to showcase the effectiveness and versatility of extracts, EOs, and phytocompounds as natural ingredients. It also delves into their ability to enhance skin health and elevate the quality of cosmeceutical products.

### Cosmeceuticals overview

Cosmeceuticals are a combination of cosmetics and pharmaceuticals. This term was coined in 1962 by Raymond E. Reed, a member of the U.S. Society of Cosmetic Chemists, and in 1984 by Dr. Albert Kligman of the University of Pennsylvania for his work on the anti-aging effects of tretinoin (Pandey et al. 2019). The Federal Food, Drug, and Cosmetic Act (FD&C Act) does not recognize this term; however, these products are widely used in the cosmetic industry using the INCI name (International Nomenclature of Cosmetic Ingredients), followed by the Latin binomial that indicates the part of the plant employed within cosmeceutical formulation (e.g., leaf, root, rhizomes, bark), and the extraction product (e.g., extract, oil) (Ferreira et al. 2021).

A cosmeceutical is a topical skin product that reduces premature aging and maintains its integrity. This product contains bioactive ingredients that provide medical benefits. However, its effectiveness is dependent on the compounds present in the final formulation (Hu et al. 2020; Zerbinati et al. 2021). According to its function, the ingredients of a cosmeceutical are classified as humectant, emollient, emulsifier, solvent antimicrobial, skin conditioning, preservative and perfume, chelating agent, surfactant, and film-forming. The cosmeceutical industry is interested in obtaining active ingredients from aromatic and medicinal plants that fulfill at least one of the functions indicated above (Hernandez et al. 2021). The challenge of this trend in cosmetology is obtaining a formulation of safe and biocompatible functional cosmetics, biodegradable, and mild to the skin (Hernandez et al. 2021; Zerbinati et al. 2021).

Skin is a pivotal organ of the body composed of lipids and proteins, which exerts the function of a protective barrier to the internal organs (Hernandez et al. 2021). The skin protects against external threats caused by sunlight, xenobiotic agents, opportunistic pathogens, and environmental pollution (Boismal et al. 2020). It is the largest, visible, and exposed organ of the human body and changes both structural and functional due to chronological and environmental aging (Rorteau et al. 2020). It reflects its general health condition and, therefore, acts as a sensory organ that allows the monitoring and evaluation of the aging processes (Strzępek-Gomółka et al. 2021; Chaiyana et al. 2021).

**Fig. 2:**
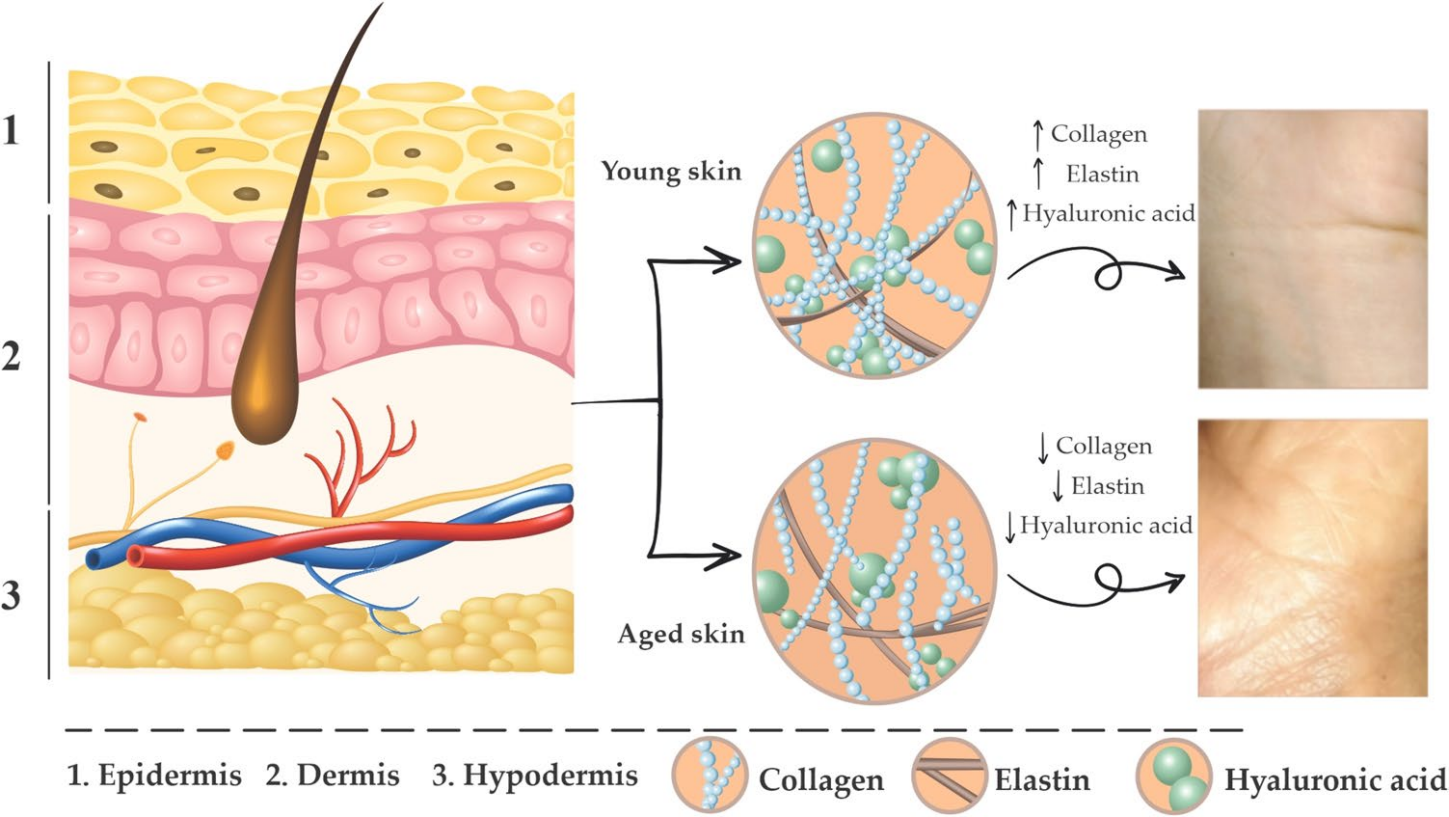
Human skin layers and main molecular components related to skin aging

At the structural level (Fig. 2), the skin consists of a complex multi-layered system that includes the epidermis, dermis, and hypodermis (Yazdi and Baqersad 2022). The epidermis is a dynamic structure that acts as a selectively permeable membrane that maintains proper moisture within the body (Lim 2021). It is the outermost layer and comprises structures known as stratum corneum, stratum lucidum, stratum granulosum, stratum spinosum, and the stratum basale. Also, it contains keratinocytes, melanocytes, mastocytes, pigment cells, Merkel cells, and Langerhans cells (Michalak et al. 2021; Yazdi and Baqersad 2022). Melanocyte cells contained in the stratum basale are responsible for synthesizing melanin in the dermis layer through the enzyme tyrosinase (a key enzyme of melanogenesis) by oxidation and hydroxylation of L-tyrosine (Carpio et al. 2021; Shanbhag et al. 2019). Melanin plays a pivotal role in heat regulation, cosmetic interaction, and camouflage. Its major function is to prevent damage caused by ROS generated by exposure to sunlight due to broadband UV absorption, as well as its antioxidants and free radical scavenging properties (Alves et al. 2020). Melanin is increased during chronic exposure to UV radiation, which results in hyperpigmentation of the skin that is observed as patches, melasma, solar lentigines, or liver spots, among others. On the other hand, the tyrosinase enzyme limits melanin production, consequently, it is the most important target for inhibitors of hyperpigmentation (Mann et al. 2018). In general, homeostasis imbalances of the epidermis are associated with skin deterioration, consequently, the epidermis is a topical important in cosmetology and related sciences. The dermis underlies the epidermis and contains fibroblasts responsible for producing collagen, elastin, and hyaluronic acid (glycosaminoglycans). Both elastin and collagen fibers are responsible for the strength and elasticity or resiliency of the skin (Strzępek-Gomółka et al. 2021; Mota et al. 2021), while hyaluronic acid with its high-water retention capacity possesses moisturizing properties. Additionally, the dermis contains numerous blood vessels and nerve endings, as well as hair follicles, sweat, and sebaceous glands (Michalak et al. 2021). The hypodermis or subcutaneous fascia is the deepest layer of skin. It is a connective tissue located under the dermis that contains both adipose lobules and skin appendages, among them hair follicles, blood vessels, and sensory neurons (Yousef et al. 2022).

**Fig. 3:**
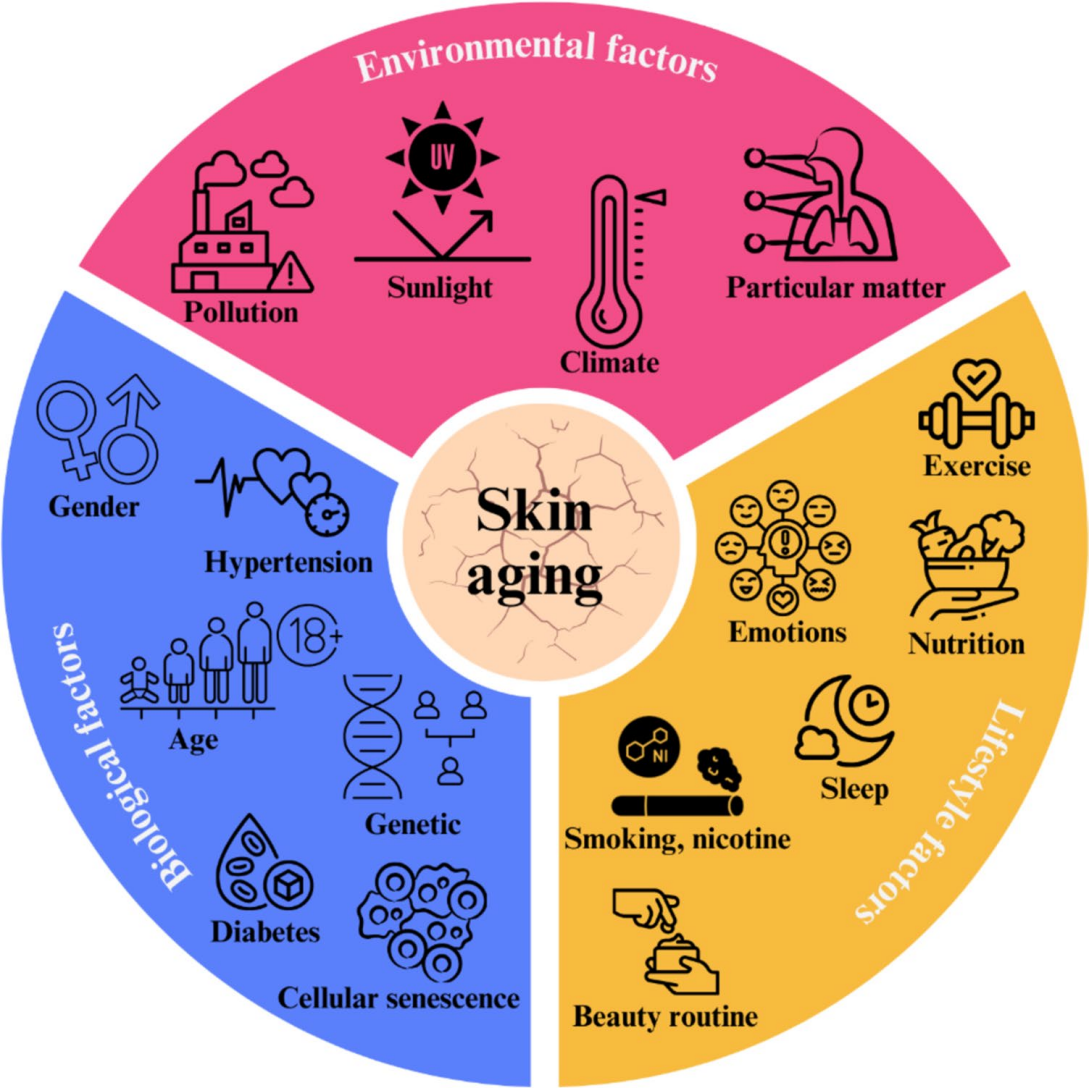
Factors associated with chronological aging and photoaging

Mechanisms leading to chronological aging of the skin are related to ROS generated during oxidative cell metabolism, DNA damage, decreased DNA repair, decreased DNA methylation and phosphorylation reaction, telomere shortening, gene loss, oncogene, and tumor suppressor gene deregulation, as well as hormonal changes, for example, the decline of estrogen (menopause) and testosterone (men and women) in old age (Orioli and Dellambra 2018; Tobin 2017). Mechanisms leading to photoaging are a consequence of chronic exposure to both UVB (290–320 nm) and UVA (320–400 nm) wavelengths. The energy transferred due to UV radiation is capable of generating ROS which leads to transcription factor activation, and lipid peroxidation, increasing ROS, MMP-1, and IL-1, among others (Ferreira et al. 2021). In general, the causes that lead to chronological aging and photoaging are categorized into three groups and are associated with the biological, environmental, and lifestyle factors of each individual (Fig. 3).

**Fig. 4:**
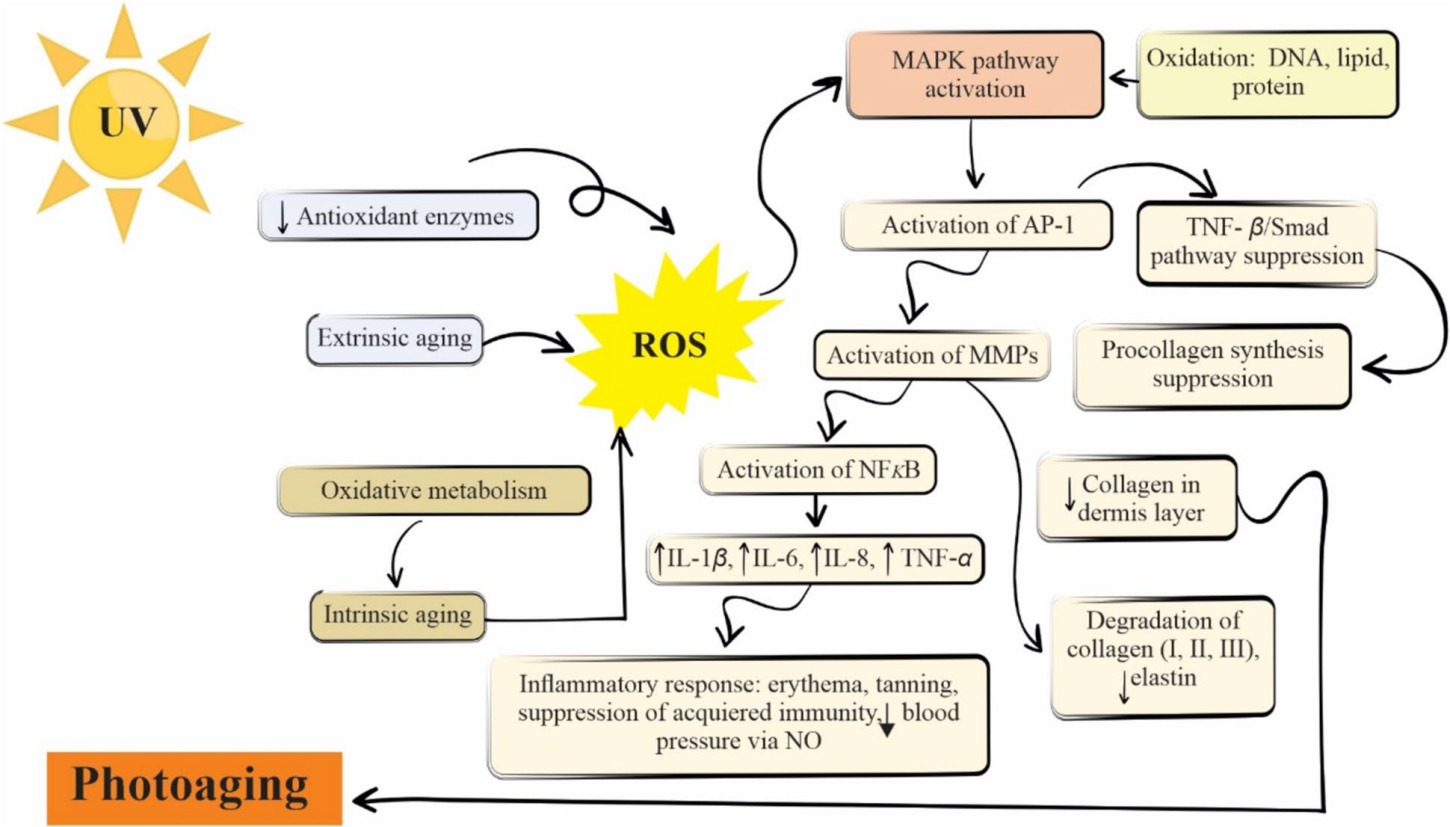
Diagram of the impact of ROS on the skin

From a molecular point of view, skin aging is mainly caused by oxidative stress due to an increase in ROS [hydroxyl (HO·), superoxide (O_2_·-), alkoxyl (RO·), peroxyl (RO_2_·), singlet oxygen (^1^O_2_) radicals, hydrogen peroxide (H_2_O_2_) and organic peroxides (ROOH)] (Shanbhag et al. 2019), which are generated by multiple factors, including oxidative metabolism, degradation of antioxidants, inflammatory response, and especially, chronic sunlight exposure (Jarzycka et al. 2013). ROS levels elevated induce the mitogen-activated protein kinase (MAPK) pathway activation. The activation of the MAPK pathway entails the activation of both the transcription factor activator protein 1 (AP-1) and the suppression of the transforming growth factor-beta/Smad signaling pathway (TGF-β/Smad), which are involved in the transcriptional regulation of several endopeptidases MMPs (matrix metalloproteinases). Both TGF-β/Smad suppression and MMPs activation are responsible for aberrant collagen homeostasis and its progressive fragmentation in the dermis layer, which accelerates chronological aging, and photoaging (Boismal et al. 2020). Thus, chronological aging is characterized by thinning epidermis and wrinkles formation, while the process of photoaging causes hyperpigmentation and deep wrinkles. Additionally, the activation of MMPs leads to inflammatory responses due to the activation of nuclear factor kappa-light-chain-enhancer of activated B cells (NFκB) regulating the transcription of proinflammatory cytokines, among them IL-1β, IL-6, IL-8, and TNF-α. In keratinocytes, NFκB activation induces both cyclooxygenase-2 (COX-2) and nitric oxide synthase (iNOS) expressions, which lead to erythema, tanning, suppression of acquired immunity, and decrease of blood pressure via nitric oxide (NO), among others (Boismal et al. 2020). A schematic representation of the impact of ROS on the skin by chronologic aging and photoaging due to intrinsic and extrinsic factors, respectively, is shown in Fig. 4.

Biological and environmental factors, as well as lifestyle habits, e.g., caloric restriction, exert an impact on healthy aging and human longevity (Zia et al. 2021). When the skin faces unfavorable conditions, it is subject to loss of its integrity, which implicates a decrease in elasticity and resistance and increases in laxity, and pigmentation processes (Zerbinati et al. 2021; Rorteau et al. 2020). Therefore, the cosmeceutical industry focuses on the design of formulations that contain active ingredients that improve the appearance of the skin and that also cross the epidermis to exert a pharmacological effect (Zerbinati et al. 2021). The strategies used include those procedures that limit the impact of aging at the epidermis, dermis, and vascular levels (Rorteau et al. 2020). Among these effects highlight photoprotective, antioxidant, anti-inflammatory, and senolytic activities, as well as inhibition of tyrosinase, melanin transfer, melanocyte proliferation, elasticity recovery, and skin hydration, which taken together, are part of anti-aging strategies (Boismal et al. 2020).

Thus, the skin’s natural aging occurs progressively as an age-related change, characterized by a decrease in its repair capacity and an increase in the production of free radicals due to intrinsic and extrinsic factors. Chronic exposure to sunlight is a critical factor since the increase in ROS levels leads to protein and DNA damage, as well as amino acid racemization and non-enzymatic glycosylation (Michalak et al. 2021). This leads to an impact on skin homeostasis which is reflected in its visual appearance. Hence, the deterioration of the epidermal and dermal layers by chronic exposure to UV radiation is known as premature aging (Hussain et al. 2021). For example, thin, pale, fair skin is due to a decrease in melanocytes, while loss of elasticities and fulfills are raised to a decrease in elastin and collagen, respectively. Other characteristics of aging include fine lines and wrinkles, sagging, hyperpigmentation, hyperkeratosis, dryness, telangiectasias, elastosis, susceptibility to irritation, decreased rate of skin regeneration, and recovery (Kusumawati et al. 2018). Under these circumstances, the skin needs anti-aging treatment to increase the production of its natural components or slow down natural loss (Michalak et al. 2021; Strzępek-Gomółka et al. 2021).

### Classification of cosmeceuticals

Currently, there is no universally accepted classification system for cosmeceuticals, leading to varied categorizations based on their intended use or specific etiological indications. These products can be grouped into several categories, including skin-lightening or depigmentation agents, sunscreens, moisturizing agents, anti-wrinkle and anti-aging treatments, scar reduction solutions, antioxidants, hair-strengthening products, and treatments for specific skin disorders (Pandey et al. 2019). Additionally, the category includes essential items like cleansers, moisturizers, and anti-inflammatory agents (Draelos 2017).

Among the valuable botanical species for the cosmeceutical industry, *Foeniculum vulgare* Mill., also known as fennel, stands out for its versatility. Its various forms—such as oil, seed, leaf extract, and fruit powder—are utilized in a range of products, including serums, toners, shampoos, conditioners, and sunscreens with sun protective factor (SPF) 50 (https://incidecoder.com/search?query=Foeniculum+vulgare+/Accessed September 01, 2024). This plant is often used in combination with other aromatic species to develop a variety of cosmeceuticals with fennel as a key component. Among the variety of fennel-based cosmeceuticals, the Lather Advanced Blemish Control serum stands out. This serum is designed for the treatment of skin blemishes and is suitable for application on the entire face or in specific areas. The formula also includes beneficial ingredients such as *Aloe barbadensis* Miller. (*A. vera* L.) leaf extract, *Achillea millefolium* Linn. root extract, and essential oils such as *Mentha piperita* L. and *Melaleuca alternifolia* (Maiden y Betche) Cheel. Together, these components contribute to the serum’s effectiveness in promoting clear, healthy skin.

**Fig. 5:**
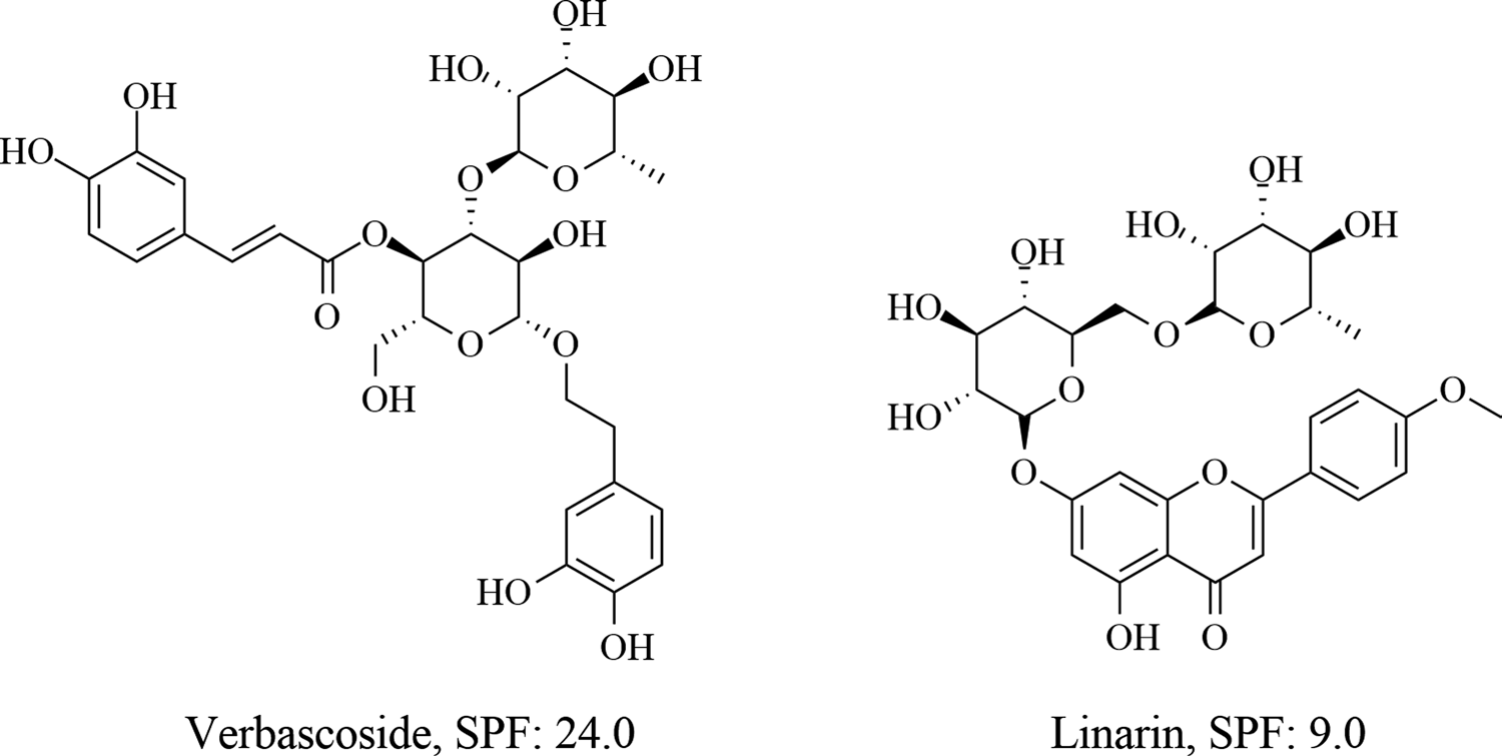
Chemical structures of verbascoside and linarin and their SPF values

In addition to extracts or EOs, phytocompounds are pivotal ingredients in the development of cosmeceuticals, offering various beneficial properties. For example, verbascoside and linarin (Fig. 5), derived from *Buddleja scordioides* Kunth (escobilla or butterfly bush), are recognized for their antioxidant and photoprotective effects. These compounds have been evaluated for their potential in formulating effective plant-based sunscreens, demonstrating promising results in protecting the skin from UV damage (Acevedo et al. 2005).

### Aromatic plants as cosmeceutical ingredients

For millennia, aromatic plants have been valued for their ability to promote healthy skin and treat various degenerative skin conditions (Ribeiro et al. 2015). Consequently, the industry increasingly utilizes their extracts, essential oils, and phytocompounds as natural alternatives, recognized for their safety, efficacy, and environmental sustainability (Alves et al. 2020).

The evaluation of plants’ chemical composition and biological effects is essential to identify their beneficial properties. Among the various natural compounds, those with antioxidant effects are particularly important for their application in the cosmeceutical sector. However, due to the increasing interest in these products, it is crucial to assess the extinction risks to promote sustainable use and the implementation of effective extraction methods (Hazrati et al. 2024). Furthermore, it is relevant to analyze the adaptability of plants to extreme conditions, such as high salinity, drought, and elevated temperatures. The species that demonstrate resilience in these challenging environments are considered valuable sources of natural antioxidants (Chekroun-Bechlaghem et al. 2019).

**Fig. 6:**
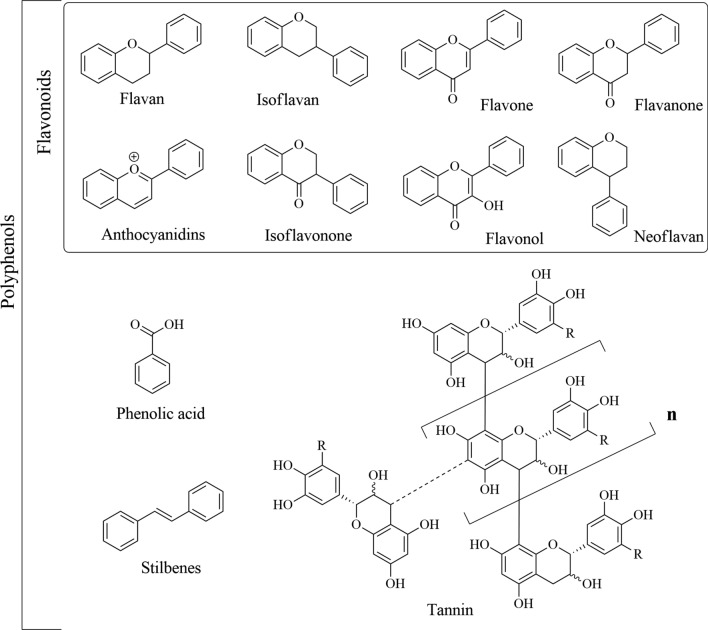
Chemical structures of some natural antioxidants

Many of the antioxidant compounds identified as cosmeceutical ingredients belong to the group of polyphenols, triterpenes, steroids, fatty acids, polysaccharides, carotenoids, and peptides, which are extracted from vegetal material with appropriate solvents (Fuentes et al. 2021; Felix-Cuencas et al. 2022). Of these molecules, noteworthy polyphenols including flavonoids, phenolic compounds, tannins, and stilbenes (Fig. 6). Flavonoids serve as enzyme cofactors and play a significant role in the angiogenic process. They exhibit a range of beneficial activities, including anti-inflammatory, anti-aging, anti-cellulite, and anti-couperose (anti-rosacea) effects. Additionally, flavonoids demonstrate moisturizing, softening, and skin-lightening properties. Phenolic compounds, particularly phenolic acids, display anti-tyrosinase activity, stimulating collagen synthesis, and elastin production. They also possess anti-allergic, anti-inflammatory, antimicrobial, anti-aging, and photoprotective properties, which help prevent UV-induced erythema. Tannins promote wound healing and exhibit antimicrobial, anti-inflammatory, anti-cancer, and anti-ulcer activities. Furthermore, they enhance tropoelastin synthesis and reduce elastase activity. Stilbenes also demonstrate anti-tyrosinase activity and influence the post-transcriptional regulation of melanogenic genes, inhibiting the mRNA expression of tyrosinase (TYR), TYR-related proteins 1 and 2, microphthalmia transcription factor, and dopachrome tautomerase in human melanocytes (Michalak 2022).

### Phytocompounds isolated from extracts with cosmeceutical properties

Recent studies highlight glabridin, a prenylated isoflavonoid from *Glycyrrhiza glabra* L. (licorice) root extract, as a key compound contributing to skin-lightening effects (Hu et al. 2020). In addition to its depigmenting activity, licorice has been reported to improve skin wound healing via angiogenesis increases and collagen deposition (Assar et al. 2021). On the other hand, isovitexin, a flavonoid derived from *Achillea alpina* L., effectively downregulates melanin synthesis, demonstrating skin-lightening potential (Shanbhag et al. 2019).

Curcumin, a traditional bioactive component widely used as a species and medicine, is a polyphenol isolated mainly from *Curcuma longa* L. rhizomes. The scientific evidence has shown that topical application and its oral intake contribute to maintaining skin integrity because it accelerates wound healing (Zia et al. 2021; Akbik et al. 2014). Therefore, it is used for acne, atopic dermatitis, iatrogenic dermatitis, eczema, psoriasis, vitiligo, photoaging, and other skin disorders (Vaughn et al. 2016; Vollono et al. 2019). This natural pigment exerts protective effects against oxidative stress caused by ROS production, peroxidation of lipids, protein carbonylation, and mitochondrial permeability transition. Other positive effects of curcumin include the regulatory role of the immune system and cellular senescence (Zia et al. 2021).

The anti-aging effects of epigallocatechin gallate (a polyphenol found in *Camellia sinensis* L. or green tea) are especially associated with wound-healing stages and its repair mechanisms, which are related to antioxidant, antimicrobial, anti-inflammatory, antifibrotic, and angiogenesis activities (Xu et al. 2021). This polyphenol protects against immune suppression and photocarcinogenesis, as well as DNA damage and erythema formation (Saewan and Jimtaisong 2015). Further, epigallocatechin gallate possesses moisturizing and anti-melanogenesis properties and decreases wrinkle-forming (Kim 2016).

Although *Vitis vinifera* L. (grapes) and *Vaccinium corymbosum* L. (blueberries) are not classified as aromatic species, their fruits possess notable sensory properties. In the cosmeceutical industry, both species are relevant. Resveratrol or trans-3,4´,5-trihydroxystilbene, is a stilbene found mainly in grapes and blueberries fruits, respectively, and has been scientifically shown to increase life expectancy after consumption, earning it the designation of a “longevity-prolonging agent” (Ortega and Duleba 2015). This compound is considered a multifunctional cosmeceutical because it exerts anti-aging properties related to its antioxidant, anti-inflammatory, anti-angiogenic, anti-bacterial against acne vulgaris and anti-cancer effects due to its capacity for inhibiting cyclooxygenases activity, modulates tyrosinase activity, and increases collagen I/II production, also inhibits expression of AP-1, MMPs, NFκB and proinflammatory cytokines such as IL-1β, IL-6, IL-8, TNF-α, and decreases chronic skin damaged by photoaging (Saewan and Jimtaisong 2015; Ratz-Łyko and Arct 2019).

*Thymus serphyllum* Linn. (thymus) and *T. vulgaris* L. (garden thyme) are two aromatic species that exert anti-elastase and anti-inflammatory activities due to containing thymol, a phenolic monoterpene that has shown anti-aging effects since inhibits elastase enzyme, TNF‐α, and IL‐6 levels, suppress iNOS and COX‐2 expression, and blocks the p38 mitogen‐activated protein kinases phosphorylation (Salehi et al. 2018).

Another class of interesting compound are phenylpropanoids. Phenylpropanoids are multifaceted compounds, including antimicrobial, antioxidant, anti-inflammatory effects, and photoprotective properties (Neelam et al. 2020). Studies have shown that hydrocinnamic acid, also called *p*-coumaric acid, obtained from ginseng leaves, inhibits tyrosinase activity and melanin content; therefore, it can be useful in hyperpigmentation treatments (Hu et al. 2020).

**Fig. 7:**
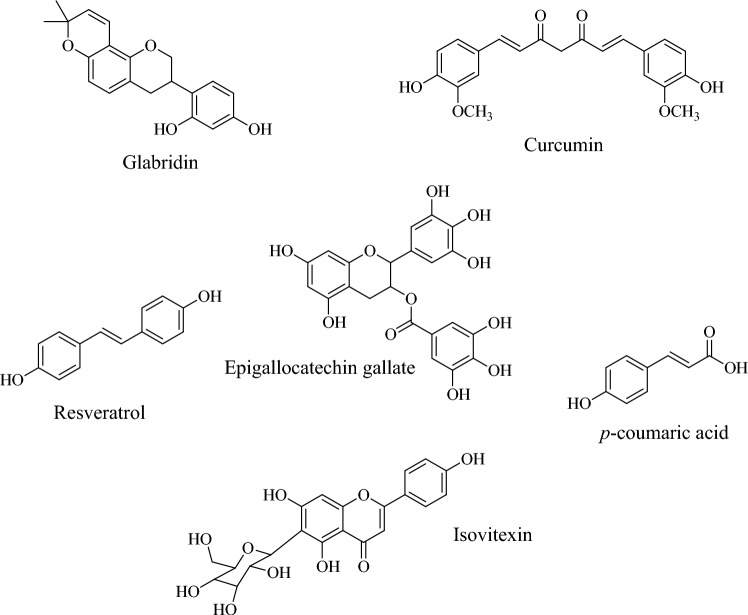
Chemical structures of some phytocompounds isolated from plant extracts with cosmeceutical properties

Chemical structures of *p*-coumaric acid, glabridin, isovitexin, resveratrol, curcumin, and epigallocatechin gallate are shown in Fig. 7.

### Phytocompounds isolated from essential oils with cosmeceutical properties

Essential oils are extensively utilized in the cosmeceutical industry due to their numerous properties that promote healthy skin, enhancing elasticity and firmness. These ingredients also possess pharmaceutical benefits, making them suitable for treating conditions such as stretch marks, acne, dark spots, and premature skin aging. Additionally, EOs serve as natural fragrances, preservatives, surfactants, and masking agents. They are primarily obtained from various plant parts, including flowers (*Cananga odorata* Hook. F. & Thomson or ylang-ylang), leaves (*Cymbopogon citratus* (DC.) Stapf or lemongrass), fruits (*Piper nigrum* L. or black pepper), peels (*Citrus reticulate* L. or tangerine), seeds (*F. vulgare* L. or fennel), berries (*Juniperus communis* L. or juniper), bark (*Cinnamomum verum* J. Presl. or cinnamon), roots (*Zingiber officinale* Roscoe or ginger), rhizomes (*Curcuma longa* L. or turmeric), and resin (*Commiphora myrrha* (Nees) Engl. or myrrh). Among the EOs of high industrial value are citrus, floral, lavender, or phyto-essences such as geraniol/nerol, linalool, citral, and citronellal (Sharmeen et al. 2021).

The studies demonstrate that appropriate concentrations of monoterpenes in EOs can benefit skin health. For example, EOs derived from the flowering aerial parts of *Helichrysum italicum* (Roth) G. Don, along with its phyto-components *α*-pinene and limonene, exhibit anti-collagenase and anti-elastase activities (Fraternale et al. 2019). Capetti et al. (2021) reported that phytoconstituents from *Cymbopogon schoenanthus* L*.*, *Litsea cubeba* (Lour.) Pers. (a recognized skin-whitening agent), *Melissa officinalis* L., and *Verbena officinalis* L. have shown anti-tyrosinase potential through bioassay-oriented fractionation. In this study, *β*-myrcene (identified in both *L. cubeba* Lour. Pers. and *V. officinalis* L. (EOs) and (+)-citronellal (from *M. officinalis* L. EO) enhance tyrosinase inhibitory activity of citral. Combinations of EOs from *Citrus reticulata* L. (tangerine), *Melaleuca alternifolia* (Maiden y Betche) Cheel (tea tree), *Eucalyptus camaldulensis* Dehnh (eucalyptus), and *Lavandula angustifolia* Mill. (lavender) help maintain hydrolipidic balance, increasing skin hydration while reducing sebum levels (Infante et al. 2022). Linalyl acetate, isolated from the extract and essential oil of *Achillea millefolium* Linn*.*, exhibits skin-lightening activity by suppressing melanin production (Shanbhag et al. 2019). Additionally, thymol has been shown to alleviate atopic dermatitis associated with *Staphylococcus aureus* (Kwon et al. 2018) and acts against *Cutibacterium acnes* (Folle et al. 2021). Thymol also mitigates dermal inflammatory infiltrates and possesses antipruritic effects (Wang et al. 2020). Furthermore, the anti-inflammatory properties of thyme EO influence leukocyte migration, which is pivotal to enhancing wound healing (Salehi et al. 2018).

**Fig. 8:**
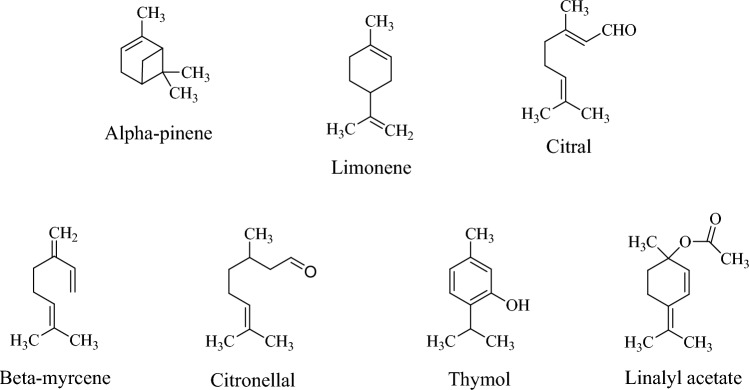
Chemical structures of monoterpenes with potential cosmeceutical properties

The chemical structures of these monoterpenes are shown in Fig. 8.

### Extracts and essential oils as photoprotective agents

Over the past decade, there has been a significant increase in interest in cosmeceuticals, particularly in photoprotective development agents as an essential strategy to mitigate the signs of aging. In alignment with industry trends, numerous researchers have focused their studies on identifying plant extracts and essential oils rich in antioxidant metabolites. These natural ingredients are being explored for their potential to reduce ROS activity and protect the skin from damage associated with chronic sun exposure. Scientific evidence supports that these ingredients can function as natural antioxidants, enhancing sun protection and photostability (Hübner et al. 2020).

The U.S. Food and Drug Administration (FDA) mandates spectrophotometric analysis for broad-spectrum photoprotective agents, with the SPF being a pivotal parameter. SPF measures the effectiveness of a sunscreen in protecting against UVB rays, with SPF 15, SPF 30, and SPF 50 offering approximately 93%, 97%, and 98% protection, respectively. This metric is essential for assessing the efficacy of antioxidant agents in photoprotective formulations, as it indicates the fraction of solar energy (UV radiation) that reaches the protected skin (Strzępek-Gomółka et al. 2021). Examples of plant extracts and essential oils studied for their photoprotective properties, along with their corresponding SPF values, are presented in Table 1.

**Table 1:** Extracts or essential oils as natural antioxidants and their sun protection factor

Plant species	Common name	Cosmeceutical properties	SPF	References
*Baccharis antioquensis* Killip & Cuatrec.	Baccharises	Photoprotective (PE)	9.10	(Mejía-Giraldo et al. 2021)
*Calendula officinalis* L.	Pot marigold	Photoprotective (EO)	8.36 14.84	(Lohani et al. 2019) (Mishra et al. 2012)
*Cistus x incanus* L.	Hairy rockrose	Photoprotective, anti-hyperpigmentation, anti-melanoma (PE)	3.33	(Gaweł-Bęben et al. 2020)
*Cistus ladanifer* L.	Common gum cistus	Photoprotective, anti-hyperpigmentation, anti-melanoma (PE)	4.37	(Gaweł-Bęben et al. 2020)
*Crataegus pentagyna* Waldst. & Kit. ex Willd.	Small-flowered black hawthorn	Photoprotective (PE)	24.47	(Ebrahimzadeh et al. 2014)
*Cymbopogon citratus* (DC.) Stapf	Lemon tea	Photoprotective (EO)	10.00	(Caballero-Gallardo et al. 2022)
*Cymbopogon flexuosus* (nees ex steud.) w. watson	Lemon grass	Photoprotective, antioxidant (EO)	13.40	(Caballero-Gallardo et al. 2022)
*Cymbopogon* sp*.*	Lemon grass	Photoprotective (PE)	6.28	(Kaur and Saraf 2010)
*Ginkgo biloba* L.	Maidenhair tree	Photoprotective, antioxidant (PE)	7.06	(Cefali et al. 2019)
*Helianthus annuus* L.	Common sunflower	Photoprotective, antioxidant, anti-inflammatory (PE)	6.30	(Banerjee et al. 2019)
*Lippia alba* (Mill.) N.E.Br. ex Britton & P.Wilson	Quick relief	Photoprotective (EO)	9.60	(Caballero-Gallardo et al. 2022)
*Lippia microphylla* Benth.	Alecrim-pimenta	Photoprotective (PE)	26.82	(Nunes et al. 2018)
*Lippia origanoides* Kunth (Carvacrol/thymol)	Wild oregano	Photoprotective (EO)	11.70	(Caballero-Gallardo et al. 2022)
*Oncosiphon suffruticosum* L.	Stinkingweed	Photoprotective (EO)	2.29	(Adewinogo et al. 2021)
*Pelargonium graveolens* L'Hér.	Sweet-scented	Photoprotective (EO)	6.45	(Lohani et al. 2019)
*Pentacalia pulchella* (Kunth) Cuatrec.	Pentacalia	Photoprotective (PE)	7.30	(Mejía-Giraldo et al. 2021)
*Ruta graveolens* L.	Rue	Photoprotective, antioxidant (PE)	5.34	(Cefali et al. 2019)
*Schinopsis brasiliensis* Engl.	Red quebracho	Photoprotective (PE)	6.00	(de Lima-Saraiva et al. 2017)
*Sphaeranthus indicus* L.	East Indian globe thistle	Photoprotective. Decreases sebum, erythema, and melanin. Increases hydration and elasticity (PE)	3.85	(Ahmad et al. 2020)
*Tagetes lucida* Cav.	Winter tarragon	Photoprotective, antioxidant (EO)	14.70	(Caballero-Gallardo et al. 2022)

Essential oil *EO* and plant extract *PE*

### Extracts and essential oils as multifunctional cosmeceutical ingredients

Extracts and essential oils derived from plants contain a complex array of bioactive phytoconstituents capable of activating multiple biological pathways. These pathways are linked to anti-aging strategies, including antioxidant, antityrosinase, antibacterial, antifungal, anti-inflammatory, and photoprotective activities, where beneficial effects on skin health can be monitored through several properties, such as hydration, skin lightening, wound healing, and rejuvenation (Shanbhag et al. 2019).

Extracts from *Panax ginseng* C.A.Mey., *Glycyrrhiza glabra* L., *Prosopis cineraria* (L.) Druce, *Cannabis sativa* L., *Sambucus nigra* L., *Camellia sinensis* L*.*, *Punica granatum* L.*,* and other aromatic plants are traditionally used to treat skin problems and their activities include the activation of different mechanisms, which are mentioned below.

*Panax ginseng* C.A.Mey. or *Ginseng ginseng*, Korean or Chinese ginseng, is a plant widely used for its multifunctional activities in skin health care and many chronic diseases (Hu et al. 2020). It is useful as an antioxidant agent and is very employed as an anti-aging strategy by the cosmeceutical industry. Its pharmacological activities include, mainly, the anti-inflammatory activity (reduces NO production and iNOS mRNA synthesis) and photoprotective effect (inhibits both COX-2 mRNA-level expression and TNF-α induced by UVB radiation) (Saewan and Jimtaisong 2015; Li et al. 2021). *Ginseng* improves replicative senescence and senescence-associated molecules, as well as melanogenesis-related proteins (Cho et al. 2021).

*Glycyrrhiza glabra* L. is a potent skin-lightening agent that inhibits tyrosinase activity and reduces UV-induced hyperpigmentation. Therefore, this species is considered a disruptor of the pigment production pathway and an anti-aging agent (Hu et al. 2020). Also, it has shown anti-inflammatory, antioxidant, anti-acne, antibacterial, antifungal, and dandruff properties, as well as improves epidermal hydration and transepidermal (Juncan et al. 2021).

*Prosopis cineraria* (L.) Druce (Khejri or golden tree” of the desert) is the most important species in the desert area of Rajasthan, as well as in Afghanistan, Saudi Arabia, and Pakistan (Pareek et al. 2015). The bark extract from golden trees induces skin rejuvenation and has been shown to exert antioxidant and antimicrobial properties. Also, it inhibits tyrosinase and lipoxygenase activities. Skin health-promoting effects include depigmentation, and decrease erythema, melanin, and sebum contents, as well as an increase in hydration and elasticity (Mohammad et al. 2018; Imhof and Leuthard 2021).

*Cannabis sativa* L. (cannabis or marijuana), and cannabinoids isolated from it, have been used as an anti-aging treatment in hair growth and dermatological disorders such as pruritus, atopic dermatitis, acne, psoriasis, lupus, eczema, nail-patella syndrome, cancer, exerting beneficial effects on skin by modulation of the endocannabinoid system (Dhadwal and Kirchhof 2018; Baswan et al. 2020; Gupta and Talukder 2021).

*Capparis spinosa* L. (caper bush) is an aromatic species that has been used especially for its moisturizing, anti-erythema properties (Saewan and Jimtaisong 2015), and melanogenesis regulation effect (Matsuyama et al. 2009). The methanolic extract from flowering buds of it exerts antioxidant activity and reduces UVB-induced skin erythema (Bonina et al. 2002).

*Solidago virgaurea* L. subsp. Alpestris (solidago or goldenrod) has demonstrated remarkable senolytic activity, i.e., its ability to target and eliminate senescent cells. Research indicates that this plant effectively reduces both senescent and papillary phenotypes, thereby improving fibroblast functionality in human skin. As a result, this species plays an important role in mitigating age-related declines in tissue functionality (Lämmermann et al. 2018).

*Allium sativum* L. (garlic) is another aromatic plant that exerts protective effects on cellular senescence and photoprotection on skin cells. This plant has showed decreased MMP-1 protein and mRNA expressions inhibit IL-1β and IL-6 levels and ameliorates senescence-associated beta-galactosidase (SA-β-gal) and Sirtuin 1 (SIRT1) activity (Kim 2016).

*Sambucus nigra* L. (elderberry) is a plant with anti-inflammatory and photoprotective properties, which has been amply used in treating skin photoaging. Its extract decreases MMP-1 expression, MAPK/AP-1, and NFκB inhibition. Also, it blocks extracellular matrix degradation, increases oxidative defense capacity by improving Nrf2/HO-1 signaling, and promotes procollagen type I synthesis because it enhances TGF-β signaling activation (Mota et al. 2021; Lin et al. 2019).

*Camellia sinensis* L. (green tea) is a potent antioxidant and anti-inflammatory agent. Water extracts from green tea are recognized for inhibiting tyrosinase activity and exerting chelating properties at its active site, also for inhibiting melanogenesis. Its anti-aging properties are related to the reduction of dark spots, wrinkles, and the effects of lightening the skin; therefore, is a powerful skin photoprotective agent (Hu et al. 2020). Green tea supplementation decreases enzyme MMP-3 production and increases elastin and collagen fibers, which explains the anti-wrinkle effect (Prasanth et al. 2019).

*Punica granatum* L. (pomegranate) is considered nowadays a promising aromatic plant with antioxidant, antimicrobial, and anti-inflammatory effects (Jalali et al. 2021), which are related to its high content of anthocyanins and hydrolyzable tannins (Saewan and Jimtaisong 2015; Zhao et al. 2021). Pomegranate extract is a UVA-photoprotective agent because it inhibits both keratinocyte proliferation and apoptosis, reducing UV damage in skin cells (Saewan and Jimtaisong 2015), also, restoring the skin glow after sunlight exposure (Michalak et al. 2021). Other important effects are related to hair care since it acts as an anti-lice and antidandruff agent (Bhinge et al. 2021). In traditional medicine, pomegranate is used in polyherbal formulations together with *Matricaria chamomilla* flowers for better wound healing results (Niknam et al. 2021).

The literature on the therapeutic benefits of aromatic plants is endless. In this review, the evidence for the cosmeceutical properties of 65 plant species gathered in 16 botanical families characterized by being highly fragrant, including Annonaceae, Apiaceae, Asteraceae, Burseraceae, Combretaceae, Cupressaceae, Lamiaceae, Lauraceae, Malvaceae, Myrtaceae, Piperaceae, Poaceae, Rosaceae, Rutaceae, Tamaricaceae, and Zingiberaceae, many of which have a long history of use in traditional medicine are summarizes in Table 2. Research-based information on phytochemistry and pharmacology allowed us to gather the beneficial properties for the skin offered by some of these plant species and their possible function within cosmeceutical formulation, noteworthy the antioxidant, anti-aging, and antiphotoaging properties, and benefits related to wound healing, skin hydration, antimicrobial, and depigmenting activities. Given the complexity of the chemical and structural composition of the compounds present in plant extracts and essential oils, it is expected that these ingredients in cosmeceutical formulations show multifunctional effects; therefore, they act on multiple targets, exerting more than one bioactivity that enhances the skincare benefits.

**Table 2:** Cosmeceutical properties observed in extractives derived from aromatic plant species

Plant species	Common name	Mechanism of action	Bioactivity	References
Annonaceae				
* Cananga odorata (Lam.) Hook.f. & Thomson*	Ylang ylang	⊥Melanogenesis	Radiance, mattifying, regenerating, antioxidant, anti-pollution	(Matsumoto et al. 2014; Georgiev et al. 2018)
Apiaceae				
* Anethum graveolens* L.	Dill	Modulator of melanogenesis	Skin whitening	(Taddeo et al. 2019)
* Angelica archangelica* L.	Angelica	⊥MMPs, ⊥Mitogen-activated protein kinase signaling pathways, ⊥collagen degradation	Photoprotective, anti-aging	(Sun et al. 2016b)
* Apium graveolens* L.	Celery	↑Skin reepithelialization, ↑fibroblast proliferation, ↑cytokeratin (CK)-17 expression, ↓percentage of the wound area, inflammatory cell infiltration, ↓bacterial colonization in skin wound tissue	Skin regeneration, atopic dermatitis, methicillin-resistant *Staphylococcus aureus* (MRSA)-associated skin infections	(Prakoso et al. 2020)
* Centella asiatica* L. Urb.	Gotu Kola	↓Oxidative damage in dermal fibroblasts, ↓MMP-9 expression, ↓breakdown of collagen by MMP-9	Antioxidant, anti-aging, anti-inflammatory, ↑collagen synthesis, stimulates the fibroblast proliferation, anti-cellulite effect, ↑epidermal barrier function	(Juncan et al. 2021; Buranasudja et al. 2021)
* Coriandrum sativum* L.	Coriander	⊥Activity of AP-1, ↓MMP-1, ↑procollagen type I	Photoprotective	(Hwang et al. 2014)
* Daucus carota* L.	Carrot seed	–	Astringent, vulnerary, anti-inflammatory, maturative, analgesic, depurative, anti-aging, tissue regenerative	(Gilca et al. 2018)
* Foeniculum vulgare* Mill.	Sweet fennel	↑Collagen, ↑elastin, ↑TGF-β1, ⊥MMPs production, ↓ROS, ↓LDH by promoting the nuclear amount of Nrf2, ↑cytoprotective antioxidants such as GSH	Photoprotective, anti-aging	(Sun et al. 2016a)
* Pimpinella anisum* L.	Aniseed	Modulator of melanogenesis	Skin whitening	(Taddeo et al. 2019)
Asteraceae				
* Achillea millefolium* Linn.	Common yarrow	↓Atopic dermatitis-like symptoms, ↓epidermal thickening, ↓transepidermal water loss	Anti-inflammatory effects on atopic dermatitis-like skin lesions, anti-allergic	(Ngo et al. 2020)
* Arnica montana* L.	Mountain arnica, mountain tobacco	–	Anti-inflammatory	(Juncan et al. 2021)
* Calendula officinalis* L.	Pot marigold	↑Collagen and non-collagenous proteins, ↑collagen organization in the initial phase of healing	Antioxidant, anti-wrinkle, skin regeneration, deep hydration, anti-aging, skin burns, anti-hyperpigmentation, photoprotective, antimicrobial, anti-inflammatory, anti-irritant, anti-psoriatic, anti-callus	(Georgiev et al. 2018; Juncan et al. 2021; Mishra et al. 2012; Aro et al. 2015))
* Eclipta prostrata* (L.) L.	False daisy	⊥Tyrosinase activity	Antioxidant, depigmenting, photoprotective, hair revitalizing	(Juncan et al. 2021)
* Helichrysum italicum* (Roth) G. Don	Immortelle	⊥Arachidonic acid metabolism and other pro-inflammatory mediators, ⊥collagenase, ⊥elastase	Anti-erythematous, photoprotective, anti-aging, regenerating, depigmenting	(Sharmeen et al. 2021; Fraternale et al. 2019; Viegas et al. 2014))
* Helichrysum odoratissimum* L.	Impepho, liquorice plant, everlasting	⊥Inflammatory skin disorder by acne vulgaris (acne), exerts anti-bacterial properties against *Cutibacterium acnes*	Anti-bacterial, anti-acne	(De Canha et al. 2021)
* Leontopodium alpinum* Cass.	Edelweiss	Positive regulation of the developmental process, programmed cell death, keratinization, and cornification forming skin barriers	Anti-inflammatory, anti-wrinkle, moisturizer, photoprotective, soothing, repairing, anti-aging	(Cho et al. 2020)
* Matricaria chamomilla* L.	Chamomile	Repairs the stratum corneum and improves the moisture contents in human skin	Anti-inflammatory, wound healing, reduces transepithelial water loss, roughness, decreases scaliness, increases smoothness, antioxidant, anti-wrinkle, anti-aging, treatment of eczema	(Juncan et al. 2021; Jadoon et al. 2015))
* Tagetes lucida* Cav.	Winter tarragon	↓ROS	Photoprotective, antioxidant	(Caballero-Gallardo et al. 2022)
* Tanacetum parthenium (L.) Schultz Bip.*	Tansy	↓ROS, ↑endogenous antioxidant, ↓DNA damage, ↑DNA repair enzymes	Photoprotective	(Martin et al. 2008)
Burseraceae				
* Boswellia serrata* Roxb.	Indian frankincense	–	Moisturizing, regenerative, antioxidant, soothing	(Georgiev et al. 2018)
* Commiphora myrrha* (Nees) Engl.	Myrrh	–	Anti-aging, energizing	(Georgiev et al. 2018)
Combretaceae				
* Combretum apiculatum* Sond*.*	Red bushwillow	↓ROS	Antioxidant	(Lawal et al. 2017)
Cupressaceae				
* Juniperus communis* L.	Juniper or ripe berries	↑Antioxidant capacity	Protective, anti-pollution, antioxidant, anti-inflammatory	(Georgiev et al. 2018)
Lamiaceae				
* Coleus Forskohlii* (Willd.) Briq.	Forskohlii, makandi or kaffir potato	↓ROS	Anti-fat, anti-edema, antioxidant, detoxifying	(Georgiev et al. 2018)
* Lavandula angustifolia* Mill.	Lavender	↑NF-κB, ↑JAK/STAT, ↑IFN-*γ*, ↑IL-17*α*, ↑IL-22, ⊥PI3K, ⊥AKT	Anti-psoriasis	(Koycheva et al. 2021)
* Lavandula stoechas* Lam.	French lavender	⊥Human red blood cells hemolysis (HRBC), ⊥hypotonicity-induced lysis of HRBC membrane	Anti-inflammatory	(Boukhatem et al. 2020)
* Marrubium vulgare* L.	White horehound or common horehound	↓ROS	Antioxidant, wound healing	(Amri et al. 2017)
* Mentha x piperita* L.	Peppermint, aerial parts	↓ROS	Soothing, antioxidant, lightening	(Georgiev et al. 2018)
* Ocimum basilicum* L.	Basil, flowering herb	↓ROS	Reduce hair loss, inhibit 5*α*- reductase activity, stimulate dermal papilla cells, anti-inflammatory	(Georgiev et al. 2018)
* Perilla frutescens* (L.) Britton	Beefsteak plant	DNA repair, activation of the MAPK/ERK pathway, and cell cycle progression to the S and G2/M phases	Anti-aging, antimicrobial, soothing, photoprotective, wound healing	(Georgiev et al. 2018; Lee and Park 2021; Kim et al. 2021))
* Plectranthus grandidentatus (Gürke)*	Big-teeth coleus	↓ROS, ⊥tyrosinase, ⊥collagenase, ⊥elastase	Antioxidant, depigmenting	(Andrade et al. 2021)
* Rosmarinus officinalis* L.	Rosemary	↓ROS, ↓STAT3 activation	Antioxidant, anti-aging, anti-wrinkle, increases elasticity, firmness, moisturization, transepidermal water loss, lightens dark spots, anti-inflammatory, photoprotective, wound healing	(Juncan et al. 2021; Yeo et al. 2019; Nobile et al. 2021)
* Thymus serphyllum* Linn.	Wild thyme	↓ROS	Antioxidant, antimicrobial, analgesic, stimulant, diaphoretic, antispasmodic, and anti-inflammatory	(Jovanović et al. 2022)
* Thymus vulgaris* L.	Thymus	↓MMP-1, ↓MMP-3, ↓IL-6, ↓phosphor-ERK, ↓ phosphor-p38, ↓ phosphor-JNK, ↑TGF-*β*1, ↑procollagen type 1, ↑GSH	Anti-wrinkle, decreases epidermal thickness, photoprotective	(Sun et al. 2017)
		Upregulated the PPAR-*γ* expression, increases adiponectin production, and stimulated the adipogenesis process	Stimulates adipogenesis, anti-aging, anti-wrinkle, expression lines, face oval remodeling	(Caverzan et al. 2021)
Lauraceae				
* Cinnamomum verum* J. Presl.	Cinnamon	Enhancing re-epithelialization and keratin biosynthesis	Wound healing	(Daemi et al. 2019)
* Laurus nobilis* L.	Laurel	↓*C. acnes*-mediated proinflammatory cytokines (IL-1*β*, IL-6, and NLRP3), ⊥NF-κB in response to *C. acnes*	Immunological disorders, anti-inflammatory	(Lee et al. 2019)
* Litsea cubeba* (Lour.) Pers.	Litsea	⊥Tyrosinase activity	Anti-aging, depigmenting	(Imhof and Leuthard 2021)
Malvaceae				
* Althaea officinalis* L.	Common marsh-mallow	Stimulated cell vitality of epithelial cells, up-regulation of genes related to cell adhesion proteins, growth regulators, extracellular matrix, cytokine release, and apoptosis	Tissue regeneration	(Deters et al. 2010)
* Azadirachta indica* A.Juss.	Neem tree	Immunomodulatory and antioxidant activities	Anti-microbial, anti-inflammatory, antioxidant, antiseptic, anti-acne	(Singh et al. 2021)
* Gossypium herbaceum* L.	Levant cotton	↓ROS	Photoprotective, protection against atmospheric pollutants and heavy metals, antioxidant	(Georgiev et al. 2018)
* Theobroma cacao* L.	Cocoa	Downregulation of inflammatory markers: IgE and chemokine.	Anti-aging, firming, regenerating, restructuring, anti-inflammatory, skin hydration, photoprotective	(Skarupova et al. 2020)
Myrtaceae				
* Melaleuca alternifolia* (Maiden y Betche) Cheel	Tea tree	–	Antioxidant, anti-inflammatory; improves skin barrier and morphologic skin characteristics; moisturizer, decreases sebum levels; broad-spectrum antibacterial, anti-acne	(Michalak 2022; Infante et al. 2022; Capetti et al. 2020))
* Melaleuca leucadendra* (L.) L.	Cajeput	↑Antioxidant capacity, ⊥COX-2 expression, ↓DNA-damage	Antioxidant, photoprotective, wound healing, antimicrobial	(Silva et al. 2020; de Assis et al. 2020))
* Myrtus communis* L.	Myrtle or true myrtle	↑Glutathione, ↑SOD, ↑CAT, tissue factor activities, nitric oxide, ↓skin malondialdehyde level	Antioxidant	(Ozcan et al. 2019)
* Syzygium aromaticum* (L.) Merr. y L.M.Perry	Clove	↑Antioxidant capacity, ↓MMP-1, ↓MMP-3, ↓IL-6, ↓p-c-fos, ↓p-c-jun, ↓NFκB, ↓IkB-*α*, ↑Nrf2, ↑HO-1, ↑NQO-1, ↑p-Smad 2/3, ↑TGF-*β*1	Anti-wrinkle, anti-thickness, antioxidant, moisturizer, photoprotective	(Hwang et al. 2018)
Piperaceae				
* Pipper nigrum* L.	Black pepper	↓ROS, ↓lipid peroxidation, ↓NFκB, ↑IκB*α*	Photoprotective	(Verma et al. 2017)
Poaceae				
* Cymbopogon citratus* (DC.) Stapf	Lemongrass	↓ROS	Anti-erythema, anti-edema, photoaging	(Costa et al. 2016)
* Cymbopogon flexuosus* (nees ex steud.) w. watson	Lemongrass	↓ROS	Photoprotective, antioxidant	(Caballero-Gallardo et al. 2022)
* Cymbopogon Martini* (Roxb.) Will.Watson	Palmarosa	⊥Growth of *T. mentagrophytes* and *T. rubrum* (above 1% of EOs), and against *M. canis* and *T. verrucosum* (4% EOs)	Anti-microbial	(Gemeda et al. 2018)
* Vetiveria zizanioides* L.	Vetiver	↓tyrosinase expression, ↓oxidative stress	Depigmenting	(Peng et al. 2014)
Rosaceae				
* Agrimonia eupatoria* L.	Hurchsteeples	Anti-inflammatory, astringent, anti-bacterial	Wound healing	(Ghaima 2013)
* Crataegus laevigata* (Poir) DC.	Midland hawthorn	Regulates MAPKs/AP-1, NFκB, and NFAT Signaling Pathways	Antioxidant, anti-inflammatory	(Nguyen et al. 2021)
* Fragaria x ananassa* Duch.	Strawberry	↓ROS, ↓NF-κB, ↓p-Iκβ*α*, TNF-*α*, IL-6, IL-1*β*, ↑Nrf2, ↑CAT, ↑HO-1. Blocks collagen destruction and inflammatory responses via NF-κB and MAPK, ↑Cellular viability, ↓UVA radiation-induced DNA	Antioxidant, anti-inflammatory, photoprotective	(Saewan and Jimtaisong 2015; Gasparrini et al. 2017)
* Prunus persica* (L.) Batsch	Peach	⊥Elastase activity, ⊥collagenase activity, ⊥tyrosinase activity	Anti-wrinkle, antioxidant	(Mostafa et al. 2021)
* Rosa damascena* Mill.	Damascus rose or rose of Castile	↓ROS, ⊥hyaluronidase, ⊥elastase, ⊥tyrosinase	Anti-aging, antioxidant, anti-melanogenic, photoprotective	(Sklirou et al. 2021)
* Rosa gallica* L.	Apothecary rose	Prevents UVB-mediated skin wrinkle formation and loss of collagen/keratin fibers, ↓UVB-induced COX-2, ↓ MMP-1	Photoprotective, anti-aging	(Jo et al. 2021)
* Rosa rugosa*	Beach rose	↓ROS, ⊥elastase activity	Anti-aging	(Chae et al. 2021)
* Rubus idaeus* L.	Red raspberry	Blocks UVB-induced MMP production, promotes type I procollagen synthesis (⊥MAPK/AP-1, ⊥NF-κβ, ↑TGF-*β*/Smad,↑Nrf2	Photoprotective, moisturizer, improves ceramides production	(Georgiev et al. 2018; Gao et al. 2018))
* Rubus fruticosus* L.	Blackberry	↓IL-6, ↓TNF-α, ↓ERK ½, ↓P38, ↓JNK ½, ↓MKK4, ↓PGE2, ↓iNOS, ↓NFκB, ↓IκB*α*	Photoprotective, anti-inflammatory	(Divya et al. 2015)
Rutaceae				
* Aegle marmelos* L.	Bilwa or bael	Keratinocyte cells migration by regulation of Akt signaling, β-catenin, and extracellular signal-regulated kinase (ERK) pathways	Wound healing	(Azmi et al. 2019)
* Citrus reticulate* L.	Tangerine	↓ROS, anti-bacterial	Wound healing	(Ishfaq et al. 2021)
* Citrus sinensis* L. Osbeck	Sweet orange	Mediates downregulation of MMP1 expression, ↑Collagen, ↑SOD, ↓ PGE2, ↓COX2, ↓JNK, ↓MDA, ↓elastin, protects against DNA cleavage, ↑antioxidant, ⊥xanthine oxidase activity, ⊥lipoperoxidative activity	Photoprotective, anti-aging	(Saewan and Jimtaisong 2015; Amer et al. 2021)
* Citrus unshiu* Marc*.*	Satsuma mandarin	⊥LPS-induced iNOS, ⊥COX-2 proteins, and their mRNAs. ↓TNF-*α*, ↓IL-6, and PGE2 secretion. Induces hyaluronic acid production and Filaggrin and SPT expression	Moisturizer, anti-inflammatory	(Kim et al. 2019)
Tamaricaceae				
* Tamarix Africana* Poir.	African tamarisk	↓ROS	Antioxidant	(Chekroun-Bechlaghem et al. 2019)
Zingiberaceae				
* Curcuma longa* L.	Turmeric	⊥NFкB, ⊥MAPK, ↓iNOS, ↓COX-2, ⊥TNF-*α*, ⊥mRNA	Anti-wrinkles, photoprotective, emotional hydration manager, modulator of brain-skin connection	(Saewan and Jimtaisong 2015)
* Zingiber officinale* Roscoe	Ginger	↓ROS, ↓COX-2, ↓iNOS, ↓IL-6, ↓IL-10, ↓TNF-*α*, ↓p-ERK, ↓p-JNK, ↓p-38, ↑Nrf2, ↑HO-1	Astringent, firming, mattifying, moisturizing, antioxidant, photoprotective	(Georgiev et al. 2018)

⊥. Inhibition. ↓. Decrease. ↑. Increase

**Fig. 9:**
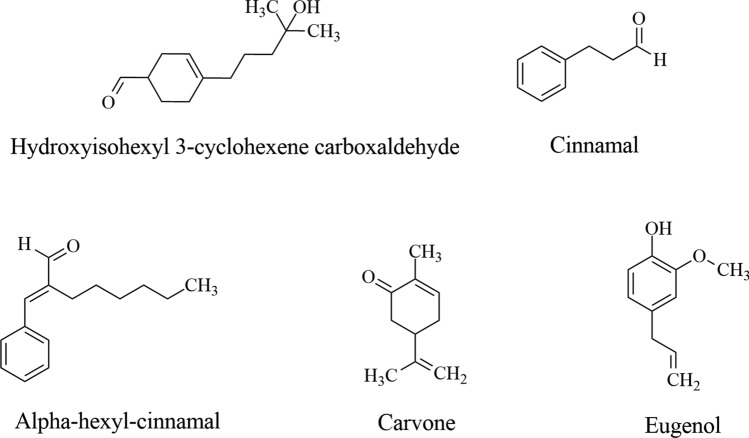
Chemical structures of natural allergens found in aromatic plant species

Although many plant-derived ingredients contribute to formulation development that improves skin health and appearance, several molecules can activate mechanisms that induce undesirable responses, manifested in allergic reactions, including contact dermatitis and phototoxicity, posing risks to human health (Ahsan 2019). Some examples of allergen molecules have been mainly identified in the Asteraceae, Brassicaceae, Lamiaceae, Lauraceae, Myrtaceae, and Rutaceae families, and belong to the coumarins, furocoumarins (Alessandrello et al. 2021), terpenes, quinones, alpha-methylene gamma-butyrolactones, phenol derivatives, and miscellaneous structures, among them isothiocyanates, disulfides, and polyacetylene derivatives (Esser et al. 2019). Some representative examples of natural allergens widely distributed in nature are hydroxyisohexyl 3-cyclohexene carboxaldehyde, cinnamal, alpha-hexyl-cinnamal, carvone, and eugenol, found in *Dipteryx odorata* (Aubl.) Willd. (Fabaceae), *Cinnamomum verum* J. Presl. (Lauraceae), *Matricaria chamomilla* L. (Asteraceae), *Mentha spicata* L. (Lamiaceae), and *Syzygium aromaticum* (L.) Merr. & L.M.Perry (Myrtaceae), respectively. Their chemical structures are shown in Fig. 9.

Finally, in response to the demand for cosmeceutical products, the industry is actively exploring and developing different innovative strategies to prevent and reverse the signs of aging (Lee 2016). The key approaches include senescent cell elimination, increasing antioxidant intake, enhancing autophagy, implementing stem cell therapies, and biotechnological strategies (e.g., chromatin organization and microRNAs) to alter plant secondary metabolism allowing for the overproduction of desired phytochemicals for cosmetic applications (Gandhi et al. 2015; Oger et al. 2019).

## Conclusions

Aromatic plants are an infinite source of bioactive molecules for the development of innovative cosmeceutical strategies. The natural antioxidants biosynthesized by plants as a defense mechanism against cell damage caused by oxidative stress are appropriate ingredients to protect human skin against the devastation of these cells due to chronological aging, which many times is accelerated by sunlight and other external factors such as weather, exposure to pollutants, lifestyle habits, among others, that taken together, accelerate skin aging causing wrinkles, spots, freckles, dehydration, loss of healing capacity, and other signs of skin aging. The scientific evidence indicates that phytochemicals and aromatic plants traditionally used as cosmetics are excellent alternatives to cosmeceutical formulations.

## Data Availability

The data presented in this study are available in manuscript.

## Abbreviations

^1^O_2_: Singlet oxygen
AKT: Serine/threonine kinase (also called protein kinase B)
AP-1: Transcription factor activator protein 1
CAT: Catalase
COX-2: Cyclooxygenase-2
CK: Cytokeratin-17
DEJ: Dermal-epidermal junction
DNA: Deoxyribonucleic acid
Eos: Essential oils
GSH: Glutathione
H_2_O_2_: Hydrogen peroxide
HO: Hydroxyl
HRBC: Human red blood cell hemolysis
IFN-*γ*: Interferon gamma
IgE: Immunoglobulin E
IL-1: Interleukin 1
IL-17*α*: Interleukin-17
IL-1β: Interleukin-1 beta
IL-22: Interleukin-22
IL-6: Interleukin 6
IL-8: Interleukin 8
iNOS: Nitric oxide synthase
JAK/STAT: Janus kinase (JAK)-signal transducer and activator of transcription (STAT) pathway
LDH: Lactate dehydrogenase
L-DOPA: L-dihydroxyphenylalanine
MAPK: Mitogen-activated protein kinase
MMP-1: Matrix metalloprotease-1
MMPs: Matrix metalloproteases
mRNA: Messenger ribonucleic acid
NFκB: Nuclear factor kappa B
NLRP3: Pyrin domain-containing protein 3
NO: Nitric oxide
Nrf2: Transcription factor nuclear factor erythroid 2-related factor 2
O_2_: Superoxide
PI3K: Phosphoinositide 3-kinase
PE: Plant extract
PPAR-*γ*: Peroxisome proliferator-activated receptor gamma
RO: Alkoxyl
RO_2_: Peroxyl
ROOH: Organic peroxides
ROS: Reactive oxygen species
SASP: Senescence-associated secretory phenotype
SA-β-gal: Senescence-associated beta-galactosidase
SIRT1: Sirtuin 1
SPF: Sun protection factor
TGF-β: Transforming growth factor beta
TNF-α: Tumor necrosis factor alpha
TRP1: Tyrosine-related protein 1
TYR: Tyrosinase
UVA: Ultraviolet A
UVB: Ultraviolet B
